# Case report: Medical cannabis—warfarin drug-drug interaction

**DOI:** 10.1186/s42238-021-00112-x

**Published:** 2022-01-10

**Authors:** Tyan F. Thomas, Evdokia S. Metaxas, Thu Nguyen, Whitni Bennett, Kathryn V. Skiendzielewski, Diane H. Quinn, Alice L. Scaletta

**Affiliations:** 1 Philadelphia College of Pharmacy, University of the Sciences, Griffith Hall 108 N, 600 S. 43rd Street, Philadelphia, PA 19104 USA; 2 Corporal Michael J. Crescenz VA Medical Center, 3900 Woodland Avenue, Philadelphia, PA 19104 USA; 3 Department of Pharmacy Practice and Pharmacy Administration, Philadelphia College of Pharmacy (PCP) at University of the Sciences (USciences), 600 South 43rd St, Philadelphia, PA 19104 USA; 4 University of the Sciences, Philadelphia College of Pharmacy, 600 S. 43rd Street, Box 34, Philadelphia, PA 19104 USA

**Keywords:** Warfarin, Medical cannabis, Anticoagulation, Drug-drug interaction

## Abstract

**Aim:**

A case of an 85-year-old patient with concurrent use of warfarin and medical cannabis containing delta-9-tetrahydrocannabinol (THC) and cannabidiol (CBD) is described. Warfarin continues to be a cornerstone of anticoagulation treatment despite the recent addition of FDA-approved anticoagulant agents. It is well known that warfarin has numerous drug interactions; however, much remains unknown about its interaction with THC and CBD. A literature review was conducted to identify documented cases of possible interactions between cannabis and warfarin. The case reports we identified noted that cannabis may potentially increase warfarin’s effect. Therefore, we aimed to determine why an effect was not seen on our patient’s warfarin dose despite daily use of medical cannabis.

**Case:**

This case report describes an 85-year-old patient who despite starting an oromucosal medical cannabis regimen of THC and CBD (which provided 0.3 mg of THC and 5.3 mg CBD once daily and an additional 0.625 mg of THC and 0.625 mg CBD once daily as needed) had minimal INR fluctuations from October 2018 to September 2019.

**Conclusion:**

Despite the introduction and use of medical cannabis therapy, with both THC and CBD components, an elderly patient with concurrent warfarin use did not see major INR fluctuations, in contrast to published literature. The potential for warfarin and THC/CBD interactions may be dependent on route of administration and dose of the cannabis product.

## Introduction

Warfarin is a widely used agent in the USA for the prevention of thrombotic complications related to atrial fibrillation and venous thromboembolism (US Department of Health and Human Services, Office of Disease Prevention and Health Promotion 2014). Due to warfarin’s narrow therapeutic index, it is paramount that health care professionals are aware of agents that may interact with it. Legalization of cannabis for both medical and recreational purposes has greatly increased its use throughout the USA. The increased use in the older population is notable, as older people are likely to be on chronic medications which may interact with cannabis, including warfarin (Han et al. 2017; Lloyd and Striley 2018). Medical cannabis is commonly used to treat chronic pain of various origins. Evidence suggests receptors in the endocannabinoid system are heavily involved in pain regulation (Lloyd and Striley 2018; Health Canada 2018). Delta-9-tetrahydrocannabinol (THC) and cannabidiol (CBD) interact with cannabinoid receptor sub-type 1 (CB1) and cannabinoid receptor sub-type 2 (CB2) receptors to produce analgesia. THC is a partial agonist at CB1&2, while CBD acts indirectly on these receptors and modulates THC’s effects (MacCallum and Russo 2018). Based on THC and CBD’s ability to inhibit cytochrome P450 enzymes CYP3A4 and CYP2C9, medical cannabis is purported to inhibit the metabolism of warfarin, increasing its anticoagulant effects. This report describes the case of an older patient on warfarin who started medical cannabis for pain management. In contrast to published literature showing INR elevation with concomitant warfarin and cannabis product use, there was no change observed in his warfarin dosing requirement despite the patient’s consistent daily use of medical cannabis for nearly a year.

## Patient case

This case report describes an older-adult patient who was maintained on warfarin therapy and later started treatment with medical cannabis for pain management. This patient, an 85-year-old, 82.55 kg male, was enrolled in our institution’s anticoagulation clinic for the management of warfarin for secondary stroke prevention in the setting of atrial fibrillation/flutter since 2012. The patient’s past medical history was significant for coronary artery disease, hypertension, hyperlipidemia, chronic obstructive pulmonary disease, stroke, and chronic lower back pain. The patient reported to our anticoagulation clinic pharmacists that he began taking medical cannabis in November 2018 to help with his chronic lower back pain. In the preceding 2 years, prior to medical cannabis use, the patient had been on a stable dose of warfarin at 22.5 mg/week with minimal deviations. The patient’s weekly warfarin doses, INR levels, and medical cannabis use are described in Table 1. In addition to warfarin, his other home medications included albuterol, amiodarone, amlodipine, atorvastatin, docusate, finasteride, folic acid, gabapentin, isosorbide mononitrate, metoprolol tartrate, and sertraline. The patient reported no relevant medication or dietary changes over the course of his concomitant treatment with warfarin and medical cannabis.

**Table 1:** Warfarin dosing, INR, and medical cannabis history

Date of anticoagulation clinic encounter	Warfarin dose (mg per week)	INR	Medical cannabis use	Other comments
October 15, 2018	2.5 mg daily except 5 mg Sundays and Wednesdays (22.5 mg/week)	2.3	None	Pt had been stable on this dose of warfarin since May 2016
November 8, 2018	22.5 mg/week	3.6-POC 3.28-Lab confirmation	None	Unclear cause for supratherapeutic INR Warfarin dose lowered to 20 mg/week
November 20, 2018	2.5 mg daily except 5 mg on Wednesdays (20 mg/week)	2.5	Started daily THC 0.3 mg/CBD 5.3 mg daily and THC 0.625 mg/CBD 0.625 mg daily as needed, November 16, 2018	No additional comments
December 6, 2018	20 mg/week	1.6	THC 0.3 mg/CBD 5.3 mg daily and THC 0.625 mg/CBD 0.625 mg daily as needed	Warfarin dose increased back to 22.5 mg/week
December 19, 2018	2.5 mg daily except 5 mg on Wednesdays and Fridays (22.5 mg/week)	2.2	THC 0.3 mg/CBD 5.3 mg daily and THC 0.625 mg/CBD 0.625 mg daily as needed	No additional comments
January 9, 2019	22.5 mg/week	2.3	THC 0.3 mg/CBD 5.3 mg daily and THC 0.625 mg/CBD 0.625 mg daily as needed	No additional comments
February 6, 2019	22.5 mg/week	2.2	THC 0.3 mg/CBD 5.3 mg daily and THC 0.625 mg/CBD 0.625 mg daily as needed	No additional comments
March 7, 2019	22.5 mg/week	2.0	THC 0.3 mg/CBD 5.3 mg daily and THC 0.625 mg/CBD 0.625 mg daily as needed	No additional comments
April 4, 2019	22.5 mg/week	1.9	THC 0.3 mg/CBD 5.3 mg daily and THC 0.625 mg/CBD 0.625 mg daily as needed	No additional comments
April 18, 2019	22.5 mg/week	2.2	THC 0.3 mg/CBD 5.3 mg daily and THC 0.625 mg/CBD 0.625 mg daily as needed	No additional comments
May 30, 2019	22.5 mg/week	2.2	THC 0.3 mg/CBD 5.3 mg daily and THC 0.625 mg/CBD 0.625 mg daily as needed	No additional comments
July 11, 2019	22.5 mg/week	2.2	THC 0.3 mg/CBD 5.3 mg daily and THC 0.625 mg/CBD 0.625 mg daily as needed	No additional comments
August 22, 2019	22.5 mg/week	1.7	THC 0.3 mg/CBD 5.3 mg daily and THC 0.625 mg/CBD 0.625 mg daily as needed	No additional comments
September 5, 2019	22.5 mg/week	2.1	THC 0.3 mg/CBD 5.3 mg daily and THC 0.625 mg/CBD 0.625 mg daily as needed	Warfarin discontinued and regimen changed to apixaban

POC, point of care INR testing

The patient reported, and his medical cannabis dispensary confirmed that he was taking a combination of two oil cannabis products administered via the oromucosal route. One of these products he self-administered once daily, every day, the other product he used as needed. His once daily product was used for basal pain control and delivered 0.3 mg of THC and 5.3 mg of CBD once daily. His product for breakthrough pain was used once daily as needed and delivered 0.625 mg of THC and 0.625 mg of CBD. Despite daily use of medical cannabis for nearly a year, his warfarin requirements remained unchanged (see Table 1). The patient’s INRs were checked consistently while the patient was taking this medical cannabis product, at intervals according to our facility’s warfarin monitoring algorithm. Further inquiry with the patient’s dispensary revealed that the patient’s medical cannabis regimen was designed to provide him with “micro-doses” of CBD and THC. This practice is based on the theory that small doses may provide minor activation of cannabinoid 1 and 2 receptors and allow the user’s body to adapt to the drug. Micro-dosing appears to be a medical cannabis industry term, as we were unable to find any published, peer-reviewed references using this term.

## Discussion

In our patient case, treatment with medical cannabis did not significantly impact warfarin therapy and INR levels remained stable. This observed effect is contrary to other reports that suggest cannabis may interact with warfarin therapy and lead to increased INR levels (Damkier et al. 2019; Yamreudeewong et al. 2009; Hsu and Painter 2019; Grayson et al. 2017; Brown et al. 2021). THC is the primary psychoactive compound present in cannabis. CBD, another major cannabinoid compound, is believed to contribute to cannabis’ therapeutic effects (Health Canada 2018; Pertwee 2014). Available dosage forms for cannabis include capsules, oils, tinctures, lozenges, edibles, topicals, rectal suppositories, and oromucosal spray (MacCallum and Russo 2018). Medical cannabis products may be prepared for oral, oromucosal, nasal, transdermal, and rectal administration (MacCallum and Russo 2018). Inhalation of the aerosols from vaporization (i.e., “vaping”) or combustion (i.e., “smoking”) are also common methods of administration (MacCallum and Russo 2018).

Our search revealed 5 relevant articles describing INR elevations in patients on warfarin who were also using cannabis products. Two case reports revealed INR elevation with smoked cannabis for recreational use (Damkier et al. 2019; Yamreudeewong et al. 2009). A third case report also showed INR elevation with primarily edible cannabis and occasional smoked cannabis use that was prescribed for anxiety and attention deficit hyperactivity disorder (Hsu and Painter 2019). A fourth case report describes INR elevation in a patient on warfarin receiving two medical cannabis sublingual oil products at a total daily dose of 14.7 mg THC and 10.3 mg CBD (Brown et al. 2021).

In addition to previously published case reports, information on the FDA-approved drug, CBD oral solution (Epidiolex®), may be helpful in predicting the effects medical cannabis may have on metabolism of concurrent drugs. The manufacturer recommends considering dose reductions of CYP2C9 substrates, such as warfarin, in patients treated with CBD oral solution. The fifth case report found that CBD oral solution, administered at a dose starting at 5 mg/kg/day and titrated up to 35 mg/kg/day, did impact warfarin therapy resulting in an elevated INR (Grayson et al. 2017).

Our patient’s medical cannabis regimen delivered between 0.064 and 0.072 mg/kg/day of CBD and did not elevate his INR. In vitro studies suggest THC and CBD both are capable of inhibiting CYP2C9 and may increase warfarin's effect in a dose-dependent manner (Health Canada 2018; Yamaori et al. 2012). We theorize that the THC and CBD amounts our patient was exposed to were lower than the amounts required to inhibit CYP2C9’s warfarin metabolism. Our theory is supported by the low ratios of the estimated maximum serum concentrations (*C*_max_) of CBD and THC and the in vitro inhibitory concentrations (*K*_i_). According to Kiyomi et al., a *C*_max_/*K*_i_ ratio < 0.1 is considered to have a low risk of causing a clinically observed drug-drug interaction (Kiyomi et al. 2004). The *C*_max_/*K*_i_ ratios for CBD and THC for this patient were 4.9 × 10^−4^ and 7.3 × 10^−4^, respectively. Because we did not have the patient’s serum CBD or THC levels, we estimated CBD and THC *C*_max_ by using dosing data reported by Miller et al. to find the best-fit line and its equation for CBD (Miller et al. 2018). Table 2 shows the CBD doses, *C*_max_, and the equation and *R*^2^ value for the best-fit trend line. Using the estimated *C*_max_, 0.87 ng/mL (by entering the patient’s daily CBD dose into the best-fit line equation), the *K*_i_ determined by Yamaori (5.6 μM), and CBD’s molecular weight (314.47 ng/nanomoles), we calculated the ratio as follows:

$${C}_{\mathrm{max}}/{K}_{\mathrm{i}}=0.87\;\mathrm{ng}/\mathrm{mL}\div 5.6\ \upmu \mathrm{mol}/\mathrm{L}=\left(0.87\;\mathrm{ng}/\mathrm{mL}\times 1000\;\mathrm{mL}\right)\div 5.6\ \upmu \mathrm{mol}=\left(870\;\mathrm{ng}\times 1\ \mathrm{nanomole}\div 314.47\;\mathrm{ng}\right)\div 5.6\ \upmu \mathrm{mol}=2.77\ \mathrm{nanomole}\mathrm{s}\div 5.6\ \upmu \mathrm{mol}=2.77\times {10}^{-3}\ \upmu \mathrm{mol}\div 5.6\ \upmu \mathrm{mol}=4.9\times {10}^{-4}$$

**Table 2:** Cannabidiol (CBD) doses, maximum serum concentrations, and best-fit trend line to estimate our patient’s maximum serum concentration

CBD dose (mg)	Maximum serum concentration (ng/mL)	Best-fit trend line and *R*^2^ value	Our patient’s estimated maximum CBD serum concentration (ng/mL)
5	0.5	*y* = 0.1476 * *x* *R*^2^ = 0.9354	0.87
10	1.1		
20	3.2		

To estimate our patient’s THC *C*_max_, we utilized the *C*_max_ and THC dosing data reported by Poyatos et al. to find the best-fit line and its equation (Poyatos et al. 2020). Table 3 shows the THC doses, *C*_max_, and the equation and *R*^2^ value for the best-fit trend line. Using the estimated *C*_max_, 0.35 ng/mL, the *K*_i_ determined by Yamaori (1.5 μM), and THC’s molecular weight (314.45 ng/nanomoles), we calculated the ratio as follows:

$${C}_{\mathrm{max}}/{K}_{\mathrm{i}}=0.35\;\mathrm{ng}/\mathrm{mL}\div 1.5\ \upmu \mathrm{mol}/\mathrm{L}=\left(0.35\;\mathrm{ng}/\mathrm{mL}\times \mathrm{1,000}\;\mathrm{mL}\right)\div 1.5\ \upmu \mathrm{mol}=\left(350\;\mathrm{ng}\times \mathrm{nanomoles}\div 314.45\;\mathrm{ng}\right)\div 1.5\ \upmu \mathrm{mol}=1.1\ \mathrm{nanomoles}\div 1.5\ \upmu \mathrm{mol}=1.1\times {10}^{-3}\ \upmu \mathrm{mol}\div 1.5\ \upmu \mathrm{mol}=7.3\times {10}^{-4}$$

**Table 3:** Tetrahydrocannabinol (THC) doses, maximum serum concentrations and best-fit trend line to estimate our patient’s maximum serum concentration

THC dose (mg)	Maximum serum concentration (ng/mL)	Best-fit trend line and *R*^2^ value	Our patient’s estimated maximum THC serum concentration (ng/mL)
5	1.6	*y* = 0.3824* *x* *R*^2^ = 0.8125	0.35
10	2.5		
20	6.7		

While our estimated *C*_max_/*K*_i_ ratios of CBD and THC were much lower than 0.1, we want to point out that the *C*_max_/*K*_i_ ratio of THC in the fourth case report by Brown et al. was 0.012, also less than 0.1, but 100 times closer to the 0.1 ratio. Despite achieving a *C*_max_/*K*_i_ ratio of less than 0.1, Brown and colleagues observed interaction between medical cannabis and warfarin (Brown et al. 2021). These conflicting findings confirm the need for additional study of the estimated plasma doses achieved with medical cannabis products and the mechanism by which medical cannabis interacts with other medications.

THC is metabolized by CYP3A4, CYP2C19, and CYP2C9, while CBD is metabolized by CYP3A4, CYP2C19, and potentially by CYP2C9 and CYP1A1/1A2 (Health Canada 2018).Though information about how THC and CBD interact with CYP enzymes varies in the literature, both THC and CBD appear to inhibit CYP3A4, CYP2C19, CYP2C9, and CYP1A1/1A2 (Health Canada 2018; Yamaori et al. 2012). Therefore, it is important that the health care professional monitors for a medical cannabis product’s potential to interact with drugs metabolized by these enzymes, which would include warfarin. Warfarin is comprised of a racemic mixture of S- and R-warfarin, and the S-enantiomer is the more potent of the two. CYP2C9 is responsible for metabolizing the more potent S-warfarin. Therefore, interactions affecting CYP2C9 metabolism are expected to have a greater effect on warfarin’s anticoagulant effects, as measured by INR levels, and the need for warfarin dosage adjustments to maintain therapeutic INR levels. Table 4 summarizes enzymes involved in warfarin, THC, and CBD metabolism.

**Table 4:** CBD and THC interactions with enzymes associated with warfarin metabolism (Health Canada 2018; Yamaori et al. 2012; Anderson and Chan 2016; Sachse-Seeboth et al. 2009)

CYP enzyme	CYP Enzyme's Role in Warfarin Metabolism	CBD and CYP Metabolism	THC and CYP Metabolism
3A4	• Metabolizes R-enantiomer	• Substrate • Inhibitor	• Substrate • Inhibitor
2C9	• Metabolizes S-enantiomer	• Potential substrate • Inhibitor	• Substrate • Inhibitor
2C19	• Metabolizes R-enantiomer	• Substrate • Inhibitor	• Substrate • Inhibitor
1A1/1A2	• Metabolizes R-enantiomer	• Potential substrate • Inhibitor	• Not a substrate • Inhibitor

Numerous variables affect the likelihood of cannabinoids, such as THC and CBD, to interact with CYP450 enzymes, including the route of administration, product formulation, pharmacogenetics, and dosage (Health Canada 2018). Cannabis products may be administered via multiple different routes, including smoking, vaping, and ingestion, and each mode of administration has a unique effect on CYP enzymes and consequently its potential to interact with drugs. For example, smoke from combustion of cannabis contains polyaromatic hydrocarbons which are capable of inducing CYP1A1/2. This could theoretically increase metabolism of R-warfarin leading to decreased INR levels. Sublingual and buccal routes are known to avoid first-pass metabolism by the liver and thus may have less potential for interactions with CYP enzymes. However, in the fourth case report, the authors describe a patient using oromucosal THC products which led to a supratherapeutic INR, suggesting that oromucosal routes do not completely avoid hepatic metabolic pathways (Brown et al. 2021). Despite the use of oromucosal medical cannabis products in our patient, we did not see any changes in INR levels that were suggestive of changes in warfarin metabolism. Therefore, we believe the THC and CBD doses consumed by our patient were below the threshold needed to produce meaningful inhibition of CYP2C9 (Health Canada 2018; MacCallum and Russo 2018; Kaminsky and Zhang 1997).

Finally, we considered the possibility of CYP activity decline as a factor for not observing an interaction between medical cannabis and warfarin. CYP-mediated phase I reactions have been shown to decline with advancing age, but phase II reactions appear to remain intact (Klotz 2009). We are unable to comment on the impact of reduced phase I metabolism on our observed findings. The link between declining CYP-mediated phase I reactions and the risk for medical cannabis-drug interactions may warrant additional investigation.

When evaluating the drug-drug interaction potential of cannabis products, one must take into consideration the route of administration and dosage of THC and/or CBD. Our report was strengthened by the frequent monitoring of the INR level and our ability to verify medical cannabis product information with the patient’s dispensary. Limitations to our clinical assessment include the following: limited information about the patient’s pain as this was primarily managed outside our facility, lack of THC and CBD serum concentrations (test unavailable at our facility, and the information was not needed to effectively manage the patient’s anticoagulation therapy), and the paucity of published data of dose thresholds of CBD and THC expected to interact with warfarin.

## Conclusion

Based on the limited studies and information available, we believe that THC and CBD used by our patient did not impact his warfarin dosing requirements because his THC and CBD doses were too low to inhibit CYP enzymes responsible for warfarin metabolism. This case provides additional evidence that THC interacts with warfarin in a dose-dependent manner. In our patient case, 0.3–0.925 mg THC and 5.3–5.925 mg CBD administered via the oromucosal route daily for up to 8 months did not impact warfarin’s metabolism or result in any significant changes in INR levels.

As legalization of cannabis continues to expand, the number of people using cannabis products will increase. Healthcare professionals must be diligent in asking about cannabis use, which includes use of CBD-only products and cannabis products (which will contain both THC and CBD compounds). If the patient confirms use, the health care professional must also attempt to determine the THC and CBD amounts in the products, the frequency of use, and route of administration. All of these considerations will help the healthcare professional to make informed decisions about the potential for drug-drug interactions. Finally, we must note that the ability to determine the THC and CBD amounts in a medical cannabis product will vary by state, as wide variability exists in state reporting requirements of THC and CBD amounts.

## Data Availability

Not applicable; no author-generated data are described in this manuscript.

## References

[CR1] Anderson GD, Chan LN (2016). Pharmacokinetic drug interactions with tobacco, cannabinoids and smoking cessation products. Clin Pharmacokinet..

[CR2] Brown GW, Bellnier TJ, Janda M (2021). Δ-9-tetrahydrocannabinol dose increase leads to warfarin drug interaction and elevated INR. J Am Pharm Assoc..

[CR3] Damkier P, Lassen D, Christensen MMH, et. al. Interaction between warfarin and cannabis. Basic Clin Pharmacol Toxicol. 2019;124(1):28-31. 10.1111/bcpt.13152 30326170

[CR4] Grayson L, Vines B, Nichol K, et. al. An interaction between warfarin and cannabidiol, a case report. Epilepsy Behav Case Rep. 2017;9:10-11. 10.1016/j.ebcr.2017.10.001 PMC5789126 29387536

[CR5] Greenwich Biosciences. Epidiolex (cannabidiol). n.d. https://www.epidiolex.com/sites/default/files/EPIDIOLEX_Full_Prescribing_Information.pdf (accessed 2020 Jan 9).

[CR6] Han BH, Sherman S, Mauro PM (2017). Demographic trends among older cannabis users in the United States, 2006-13. Addiction..

[CR7] Health Canada. Information for health care professionals: cannabis (Marihana, Marijuana) and the cannabinoids. Canada: Government of Canada (October 2018). https://www.canada.ca/content/dam/hc-sc/documents/services/drugs-medication/cannabis/information-medical-practitioners/information-health-care-professionals-cannabis-cannabinoids-eng.pdf (accessed 2020 February 11)

[CR8] Hsu A, Painter NA (2019). Probable interaction between warfarin and inhaled and oral administration of cannabis. J Pharm Pract..

[CR9] Kaminsky LS, Zhang Z (1997). Human P450 metabolism of warfarin. Pharmacol Ther..

[CR10] Kiyomi I, Brown HS, Houstn JB (2004). Database analyses for the prediction of *in vivo* drug-drug interactions from *in vitro* data. Br J Clin Pharmacol..

[CR11] Klotz U (2009). Pharmacokinetics and drug metabolism in the elderly. Drug Metab Rev..

[CR12] Lloyd SL, Striley CW (2018). Marijuana use among adults 50 years or older in the 21st century. Gerontol Geriatr Med..

[CR13] MacCallum CA, Russo EB (2018). Practical considerations in medical cannabis administration and dosing. Eur J Intern Med..

[CR14] Miller SA, Stone N, Yates, AS, et al. A systematic review on the pharmacokinetics of cannabidiol in humans. Front Pharmacol*.* 2018;9 – article 1365. 10.3389/fphar.2018.01365 10.3389/fphar.2018.01365 PMC6275223 30534073

[CR15] Pertwee R. ed. Handbook of cannabis. Oxford University Press; 2014. Available at: http://www.oxfordscholarship.com/view/10.1093/acprof:oso/9780199662685.001.0001/acprof-9780199662685. Published online January 2015 (accessed 2019 Sep 11).

[CR16] Poyatos L, Perez-Acevedo AP, Papseit E, et al. Oral administration of cannabis and delta-9-tetrahydrocanniabinol (THC) preparation: a systematic review. Medicina*.* 2020;56(309); 10.3390/medicina56060309 10.3390/medicina56060309 PMC7353904 32585912

[CR17] Sachse-Seeboth C, Pfeil J, Sehrt D (2009). Interindividual variation in the pharmacokinetics of Delta9-tetrahydrocannabinol as related to genetic polymorphisms in CYP2C9. Clin Pharmacol Ther..

[CR18] US Department of Health and Human Services, Office of Disease Prevention and Health Promotion. National Action Plan for Adverse Drug Event Prevention*.* Washington, DC: US Department of Health and Human Services; 2014.

[CR19] Yamaori S, Koeda K, Kushihara M (2012). Comparison in the in vitro inhibitory effects of major phytocannabinoids and polycyclic aromatic hydrocarbons contained in marijuana smoke on cytochrome P450 2C9 activity. Drug Metab Pharmacokinet..

[CR20] Yamreudeewong W, Wong HK, Brausch LM, Pulley KR (2009). Probable interaction between warfarin and marijuana smoking. Ann Pharmacother..

